# A new species of the genus Epidamaeus (Acari, Oribatida, Damaeidae) from China

**DOI:** 10.3897/zookeys.119.1629

**Published:** 2011-07-15

**Authors:** Lixia Xie, Maofa Yang, Rong Huang

**Affiliations:** Institute of Entomology, Guizhou University; The Provincial Key Laboratory for Agricultural Pest Management of Mountainous Region, Guiyang, Guizhou, P.R. China, 550025

**Keywords:** *Epidamaeus*, new species, checklist, distribution

## Abstract

The oribatid mite genus *Epidamaeus* Bulanova-Zachvatkina, 1957 from China is reviewed, and a list and key to all known species from China are provided. A new species, *Epidamaeus conjungenus* **sp. n.** is identified, and its morphological descriptions and illustrations are also given. The distinct characteristics of *Epidamaeus conjungenus* **sp. n.** is the coterminous ridge connected to the base of the notogastral setae. Pseudanal setae undulating attenuate, the proximal half with obvious, thorn-like barbs, the distal half smooth.

## Introduction

Oribatid mites of the genus *Epidamaeus* Bulanova-Zachvatkina, 1957 are known to be very diverse throughout the Northern Hemisphere, especially in Europe and North America (Bayartogtokh 2004). Most species of this genus inhabit the litter of forests, mosses, decaying woods and organic soil layers, and primarily feed on decomposer or plant pathogenic fungi, therefore, they play an important role in regulation of the density of plant harmful fungi (Bayartogtokh 2000). *Epidamaeus* shows high taxonomic diversity in Europe and some parts of Asia. The genus comprises more than 70 species, that cosmopolitan distributed (Subías 2011). Up to now, only 6 valid species: *Epidamaeus grandjeani*, *Epidamaeus cincinnatus*, *Epidamaeus elegantis*, *Epidamaeus longispinosus*, *Epidamaeus yunnanensis*, *Epidamaeus alticola* from China were described (Wen 1990, Wang and Norton 1993, Enami et al. 1994, Wang and Cui 1996a, Chen et al. 2010), but the collection material from different vegetation zones of the country revealed species-richness as that in the other parts of the Palaearctic and Oriental Region. This may be related to the dynamic history of the area, which had a different climate and biota found throughout China.

In the present paper, a new species *Epidamaeus conjungenus* sp. n. were described from Henan Provinces. In addition, a list and key to all known Chinese species were present.

## Material and methods

Measurements and descriptions are based on specimens mounted in temporary cavity slides that were studied using a light microscope equipped with a drawing attachment. Body length is measured in lateral view, from the tip of the rostrum to the posterior edge of the ventral plate. Length of leg segments, including the portion inserted into the next segment, is measured in the lateral aspect. The number of specimens measured does not always equal the number of specimens examined because structures are sometimes indiscernible under the circumstances where the specimens are not slide-mounted.

Terminology generally developed by Grandjean (1960) as applied by Norton (1979). All type specimens and other material studied are kept in Oudemans’ fluid and deposited in the Institute of Entomology, Guizhou University, Guiyang, China (GUGC).

## Taxonomy

### Epidamaeus

Bulanova-Zachvatkina, 1957

http://species-id.net/wiki/Epidamaeus

#### Type species:

*Oribata bituberculata* Kulczynski, 1902 (Bulanova-Zachvatkina 1957a)

#### Diagnosis.

Body reddish brown, medium sized, light to dark. propodolateral apophyses *P* mostly absent, but rarely present; the formation of tubercles of Prodorsum varied: usually only *Ba* or *Da* present, 3 pairs of tubercles (*Ba*, *Bp*, *Da*) is rare present as *Damaeus*; *La* tubercle can also be present, but weak and indistinct in dorsal view, and never in combination with *Ba* (as in *Kunstidamaeus*); *E*2a and *E*2b missing, *Va* and *Vp* rarely present occasionally. *SS* usually bristle-shaped, in shorter than ss (to 3/4 as long), bristle-shaped, mostly thin. Spinae adnata (*Sa*) usually present (subgen. *Epidamaeus*) or absent (subgen. *Akrodamaeus*); The length of legs medium to long; Tibiae I-IV without setae *d*; Setal mostly formula of genua I-IV: 4-4-3-3, rarely 4-4-4-4; Associated setal (setae *d*) formula of genua I-IV: 1-1-1-0; Setal formula of trochanters I-IV: l-1-2-1; Additional ventral seta *v*2” on I and IV missing. 6 g, 1 ag, 2 on, 3 ad. (Weigmann 2006)

#### Distribution.

Palaearctic, Oriental, Nearctic, Neotropical and Australian regions.

**Species of genus** Epidamaeus **from China**

E. alticola **Wang & Cui, 1996**

*Epidamaeus alticola* Wang and Cui 1996a: 321; 1996b: 258.

Material examined.5♂♂, 4♀♀, China, Qinghai Prov., Xining City, Huzhu National Geological Park(36°57'11"N, 102°28'55"E), from litter under Pteridophytes, 2384 m a.s.l., 23 August 2009, coll. Lixia Xie (GUGC)

Distribution. China (Qinghai).

E. cincinnatus**Wang & Norton, 1993**

*Epidamaeus cincinnatus* Wang and Norton 1993: 312; Wang et al. 2000: 323.

Material examined.11♂♂, 6♀♀, China, Hebei Prov., Chengde City, Mt. Wuling (40°36'50"N, 117°28'57"E), from litter under birch, 1362m a.s.l., 25 August 2010, coll. Lixia Xie (GUGC)

Distribution. China (Beijing).

E. elegantis**Wang & Norton, 1993**

*Epidamaeus elegantis* Wang and Norton 1993: 316-318; Wang et al. 2000: 311.

Material examined.6♂♂, 3♀♀, China, Fujian Prov., Wuyishan City, Mt. Wuyi (27°45'19"N, 118°02'56"E), from litter under the chestnut trees , 278m a.s.l., 4 August 2008, coll. Zehong Meng (GUGC)

Distribution. China (Fujian, jiangxi).

E. grandjeani **Bulanova-Zachvatkina, 1957**

*Epidamaeus grandjeani* Bulanova-Zachvatkina 1957b: 1794-1796; Wen 1990: 119; Wang et al. 2000: 256.

Distribution. China (Jilin), Russia (Tatarstan).

E. longispinosus **Wang & Norton, 1993**

*Epidamaeus longispinosus* Wang and Norton 1993: 314-316; Wang et al. 2000: 310–311.

Material examined.4♂♂, 5♀♀, China, Fujian Prov., Wuyishan City, Mt. Wuyi (27°45'24"N, 118°02'46"E), from litter of coniferous forest, 263m a.s.l., 5 August 2008, coll. Zaihua Yang (GUGC)

Distribution. China (Fujian, Jiangxi).

E. yunnanensis**Enami, Aoki & Hu, 1994**

*Epidamaeus yunnanensis* Enami et al. 1994: 43–46; Aoki et al. 2000: 6.

Material examined.2♂♂, 3♀♀, China, South of Guizhou Prov., Maolan National Nature Reserve (25°19'26"N, 107°55'59"E), from litter under Podocarpus, 819m a.s.l., 16 Sep. 2007, coll. Zaihua Yang (GUGC); 7♂♂, 6♀♀, China, Yunnan Prov., Dali City, Mt. Cang (25°38'38"N, 100°09'53"E), from litter under the pine, 1950m a.s.l., 18 December 2008, coll. Yi Yan (GUGC)

Distribution. China (Yunnan, Guizhou).

#### Key to species from China.

**Table:** 

1	Propodolateral apophysis (*P*) present, having tubercles (*La*)	*Epidamaeus yunnanensis* Enami, Aoki & Hu
–	Propodolateral apophysis (*P*) absent, not having tubercles (*La*)	2
2	Notogastral setae cincinal, genital seta g6 far from g5	*Epidamaeus cincinnatus* Wang & Norton
–	Notogastral setae not cincinal, genital setae normal for genus	3
3	Sensillus (*ss*) aciculiform, aggenital seta (*ag*) lies between Anal aperture and genital aperture	*Epidamaeus elegantis* Wang & Norton
–	Sensillus (*ss*) rod-like or flagellate, aggenital seta (*ag*) normal for genus	4
4	Sensillus (*ss*) rod-like, seta *c*1 longer than other notogastral setae	*Epidamaeus alticola* Wang & Cui
–	Sensillus (*ss*) flagellate, seta *c*1 not longer than other notogastral setae	5
5	Notogastral setae leafy, Spinae adnatae rod-like	*Epidamaeus grandjeani* Bulanova-Zachvatkina
–	Notogastral setae not leafy, Spinae adnatae not rod-like	6
6	Coterminous ridge connected to the base of the notogastral setae; Spinae adnatae not long and spinous	*Epidamaeus conjungenus* sp. n.
–	Coterminous ridge not connected to the base of the notogastral setae; Spinae adnatae long and spinous	*Epidamaeus longispinosus* Wang & Norton

### Epidamaeus conjungenus sp. n.

urn:lsid:zoobank.org:act:7B87D6F6-BE4F-408C-8451-A0500B9F4A27

http://species-id.net/wiki/Epidamaeus_conjungenus

Fig. 1 

#### Material examined.

Holotype (female in Oudemans’ fluid ), China: Luoyang city, Mt. Baiyun (34°23'25.18"N, 111°01'23.15"E), Henan province, from litter, 2100 m a.s.l., 16 Aug. 2008, coll. Li-xia Xie. Paratypes. Five adults (2 males, 3 females), with same data as holotype.

#### Etymology.

The specific name “*conjunctus*” is from Latin, and refers to the conjunct ridge.

#### Diagnosis.

Prodorsal tubercles *Da, Ba, Bp* present. Sensillus smooth, short, with conspicuous bars. *Sa* triangular, long and acuminate. *Sp* small, triangular. Enantiophysis *E2* and *V* present. *Vp* bearing seta 3*b*. The setae of notogaster acuminate, radially directed. Leg setation as follows, femora 7-6-4-4; genua 4-4-3-2; tibiae 4-4-3-3; tarsi 21-18-18-15.

#### Dimensions.

Body length 913 (holotype), 913- 932 (mean 924, 6 paratypes); body width 605 (holotype), 602- 623 (mean 610, 6 paratypes). Males slightly smaller than females: body length of males holotype and 2 paratypes) 886- 902 (mean 894), body width of males 584- 592 (mean 588); body length of females (4 paratypes) 906- 914(mean 910), body width of females (4 paratypes) 596- 624 (mean 610).

**Integument.** Microtuberculate on all enantiophyses and apophyses, rostrum, lateral prodorsum and around leg acetabula. Cerotegument granules, thick, dense on most of body and legs, except digital part of tarsi. Notogaster with exuvial scalps, legs segments and lateral part of body with dense fungus micelles and adherent debris.

**Prodorsum.** Tubercles *Da* and *Bp* well developed, broadly rounded; *Ba* represented as high ridge. Propodolateral apophysis (*P*) absent. A ridge presents the side of prodorsum. Setae *ro* (175–183 μm) and *le* (216–221 μm) long, smooth, with conspicuous barbs; mutual distance of pairs *le* slightly less than that of *ro* (1.0:1.1). Interlamellar setae (96–104 μm), dark brown, with small barbs. Exobothridial setae (94–98 μm) smooth, relatively tenuous, attenuate. Sensillus (225–232 μm), with conspicuous barbs, undulating attenuate. Comparative length of prodorsal setae: *ex* < *in* < *ro* < *le* < *ss*.

**Notogaster.** Almost circular, slightly longer than wide. Anterior and posterior margins broadly rounded in dorsal view. Spinae adnatae large, directed anterolaterad in dorsal view, distance between their bases approximately equal to that between tubercles *Bp*. Notogastral setae of *c*-, *l*- and *h*-series inserted on distinct tubercles. Setae relatively smooth, brown, acuminate. Comparative length: *lm* < *lp* < *la* = *h3* < *h2* < *h1*= *c1* = *c2* . The respective lengths: 88–94μm, 107–110 μm, 137–142 μm, 147–154 μm and 167–172 μm. Setae *c*1*, c*2 and *la* directed anterodorsad, other setae radially directed. A conjoint ridge connected to base of all notogastral setae. Mutual distance of setae *c*2 twice that of *c*1. Pseudanal setae undulating attenuate, the proximal half with obvious, thorn-like barbs, the distal half smooth. Comparative length: *ps*1> *ps*2 > *ps*3.

**Ventral region.** Epimere I with medial pit and associated groove. Enantiophyses *E2* and *V* well developed, broadly triangular in ventral view. Tubercle *Vp* bearing epimeral seta 3*b*. Parastigmatic tubercle *Sa* long, acuminate and triangular. *Sp* triangular, distinct in ventral view. Length of lateral aspect *Sp* twice as broad as *Sa*. Discidium acuminate, smaller than *Sp*, directed posterolaterad. Ventral setae faintly barbed. Setae 3*c*, 4*d* very long, flagelliform. Epimeral setation: 3-1-3-4. Anogenital region normal, seta *ad3* close to anal valves. Fissure *iad* minute, represented by small, inconspicuous pore in lateral corner of valve. Anal aperture appreciably equal to genital aperture.

**Gnathosoma**. Infracapitular mentum slightly wider than long, without noticeable microtubercles. Hypostomal setae *a*, *h* and *m* thin, slightly barbed; seta *a* relatively short. Chelicera rather strong, fixed and movable digits with three blunt teeth; setae *cha* and *chb* conspicuously barbed. Palpal setation: 0-2-1-3-8 including solenidion ω.

**Legs.** Relative lengths (I-IV): 1: 0.84: 0.95: 1.1. Leg IV 1.1 times ventral body length; Femur IV 1.44 times length of trochanter IV. Formulae of leg setation and solenidia: I (1-7- 4- 4- 21) [1-2-2], II (1-6-4-4-18) [1-1-2], III (2-4-3-3-18) [1-1-0], IV (1-4-3-3-15) [0-1-0]; Each solenidion on genu I - III coupled with a respective seta *d*, seta *d* longer than solenidion on genu I-III. Solenidion *φ*1 on tibia I flagelliform, and 2.2 times longer than *φ*2.

#### Distribution.

Known only from the type locality.

#### Remarks.

*Epidamaeus conjungenus* sp. n. can be readily distinguished from most of known species of *Epidamaeus* by the coterminous ridge connected to the base of the notogastral setae. Pseudanal setae undulating attenuate, the proximal half with obvious, thorn-like barbs, the distal half smooth. Parastigmatic tubercle *Sa* very long, acuminate, Discidium(*di*) acuminate. The strong Spinae adnatae (*Sa*). The Prodorsum of this new species is somewhat similar to *Epidamaeus verrucatus* described by >Enami and Fujikawa (1989), but the setae of notogaster of new species are smooth, and lack of Propodolateral apophysis (*P*) and present tubercles *Da* and *Bp*.

**Figure 1.**
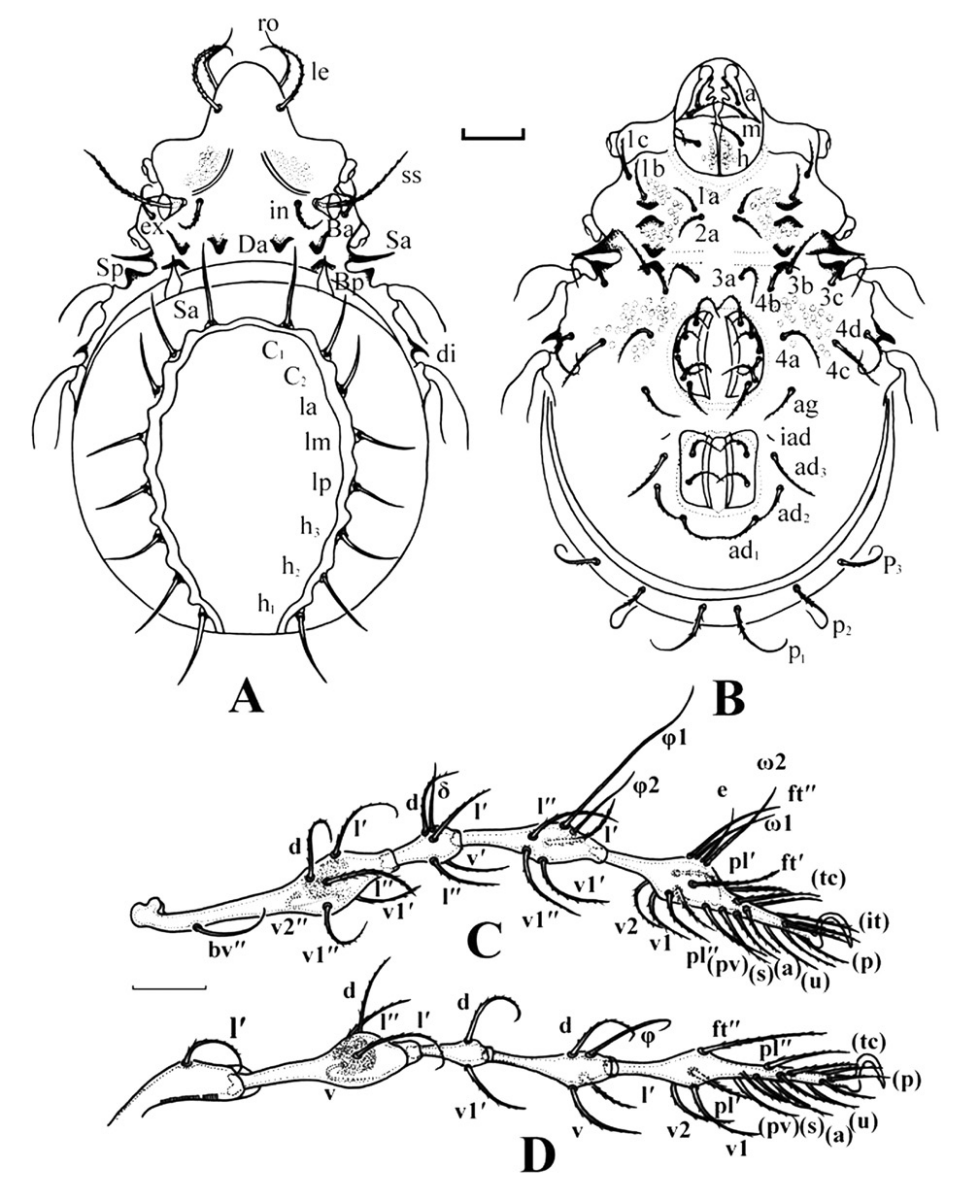
*Epidamaeus conjungenus* sp. n. **A** adult, dorsal view (100 μm) **B** adult, ventral view (100 μm) **C** leg I (100 μm) **D** leg IV (100 μm).

**Table 1.** Leg setation and solenidia of *Epidamaeus conjungenus* sp. n.

Legs	Trochanter	Femur	Genu	Tibia	Tarsus
I	v	d, l’, l’’, v1’ v1’’ bv’’, v2’’	d, σ, l’, (v)	φ1, φ2, l’, l’’, (v)	ft’, ft’’, pl’, pl’’, (v), ω1, ω2, ε, (pv), (tc), (it), (p), (u), (a), s
II	v	d, l’, l’’, bv’’, (v)	d, σ, l’’, (v)	φ, (l), (v)	ft’, ft’’, (v), ω1, ω2, (pv), (tc), (it), (p), (u), (a), s
III	l’, v’	d, l’, ev’, v’	d, σ, l’, v’	d, φ, l’, v’	ft, v’, pv’’, pv’, (tc), (it), (p), (u), (a), s
IV	v	d, l’, ev’, v’	d, l’, v’	φ, l’, (v)	ft’’, (v), (pv), (tc), (p), (u), (a), s

## Supplementary Material

XML Treatment for Epidamaeus

XML Treatment for Epidamaeus conjungenus
